# Non-Destructive Testing Using Eddy Current Sensors for Defect Detection in Additively Manufactured Titanium and Stainless-Steel Parts

**DOI:** 10.3390/s22145440

**Published:** 2022-07-21

**Authors:** Heba E. Farag, Ehsan Toyserkani, Mir Behrad Khamesee

**Affiliations:** Department of Mechanical and Mechatronics Engineering, University of Waterloo, Waterloo, ON N2L 3G1, Canada; he2farag@uwaterloo.ca (H.E.F.); ehsan.toyserkani@uwaterloo.ca (E.T.)

**Keywords:** magnetic sensors, additive manufacturing, defects, eddy current, magnetic coil, non-destructive testing, sensor designs, absolute probe

## Abstract

In this study, different eddy-current based probe designs (absolute and commercial reflection) are used to detect artificial defects with different sizes and at different depths in parts composed of stainless-steel (316) and titanium (TI-64) made by Laser Additive Manufacturing (LAM). The measured defect signal value using the probes is in the range of (20–200) millivolts. Both probes can detect subsurface defects on stainless-steel samples with average surface roughness of 11.6 µm and titanium samples with average surface roughness of 8.7 µm. It is found the signal reading can be improved by adding a coating layer made of thin paper to the bottom of the probes. The layer will decrease the surface roughness effect and smooth out the detected defect signal from any ripples. The smallest subsurface artificial defect size detected by both probes is an artificially made notch with 0.07 mm width and 25 mm length. In addition, both probes detected subsurface artificial blind holes in the range of 0.17 mm–0.3 mm radius. Results show that the absolute probe is more suitable to detect cracks and incomplete fusion holes, whereas the reflection probe is more suitable to detect small diameter blind holes. The setup can be used for defect detection during the additive manufacturing process once the melt pool is solidified.

## 1. Introduction

The way parts made by additive manufacturing (AM) technology, where materials are laid layer-by-layer, exhibits different types of defects such as cracks and porosities. There are many physical phenomena such as keyhole, solidification cracking, oxidization, lack of fusions, etc., during AM processes that cause porosities and cracks to form. The existence of these defects will affect the quality of AM-made products. To ensure the parts quality, integrity and reliability, non-destructive testing (NDT) should be carried out on these parts for early detection of defects. There is a wide range of NDT techniques that can be used in testing of AM-made parts. For example, acoustic emission testing, thermography and photo-diode signal can be used for in-situ inspection in AM [1,2]. Some other non-destructive imaging techniques such as infrared thermography and laser reflection technique were investigated in [3]. Each of these NDT techniques has its pros, cons, and challenges during the testing process. In addition, techniques such as Eddy current testing, X-ray, laser ultrasonic testing, and visual inspection can be used for the post process NDT inspection. There are different classes of AM processes. The focus of this paper is the powder bed fusion (PBF) process. In PBF, powder is sintered either by an electron or a laser beam. The types of materials used in PBF are ferrous alloys, nylon, titanium alloys, aluminum, etc. Non-destructive testing techniques such as eddy current testing, radiography and ultrasonic testing are all suitable for in-situ and post process inspection of parts made by PBF. The focus in this paper is using eddy current technique to detect subsurface defects in parts made by PBF technology. There are different challenges with using eddy current technique for defect detection in PBF [4] such as detecting unwanted signal which sometimes makes it difficult to interpret the measured signal.

**Figure 1:**
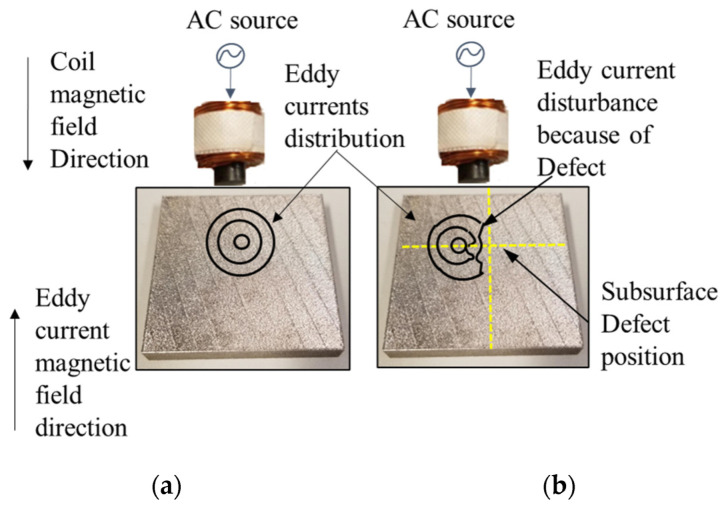
Schematic Eddy currents distribution under a coil on top of a stainless-steel plate; (**a**) shows the distribution of eddy currents in case no defect, (**b**) shows the perturbation of eddy currents due to an artificial surface crack in the plate.

Eddy current (EC) follows the electromagnetic induction law. Once a conductive material is subjected to a changing electromagnetic field which is originally created by passing an alternating current through a coil, eddy currents will start to flow in this part in a circular path or a loop as shown in Figure 1. An additional secondary magnetic field (MF) is produced by the eddy currents induced inside the material. The secondary magnetic field will oppose the primary magnetic field created in the coil of the testing probe (Lenz’s law) [5,6], and the total effect is a reduced magnetic flux linking the coil. A sensor is used to measure the total MF near the part under test [5]. The existence of any crack or defects will perturb the distribution of the eddy currents field causing variations in the phase and the magnitude of it. These variations can be measured using another receiver coil [5].

Eddy current NDT technique is used for inspection in the nuclear [7], inspection of the heat exchanger, bolt holes inspection, pipe inspection, testing of materials in aircraft energy, transportation, and aerospace industries [8], and in high-speed rails. Eddy current testing is applicable for any conductive material developed by any conventional or AM technologies. The potential applications of eddy current non-destructive testing are:

- Conductivity Testing—The ability of eddy current testing to measure conductivity can be used to identify and sort ferrous and nonferrous alloys, as well as to verify heat treatment.
- Surface Inspection—Eddy current can easily detect surface cracks in machined parts and metal stock. Inspection of the area around fasteners in aircraft and other critical applications falls under this category.
- Detection of Corrosion—Eddy current instruments can detect and quantify corrosion on the inside of thin metals such as aluminum aircraft skin [8].
- Bolt Hole Inspection—Cracking inside bolt holes can be detected using bolt hole probes, which are frequently used in conjunction with automated rotary scanners.
- Tubing inspection—Common eddy current applications include in-line inspection of tubing during the manufacturing process as well as field inspection of tubing such as heat exchangers [7].

There are some advantages and disadvantages for using the EC testing method [4,9,10]. It can be used to test parts with complex geometries where Eddy currents can go through surface coating and allows detection for subsurface defects. Advantages of this study, compared to other NDT methods are listed in [11,12]. The designed probe and the testing devices are portable because of the way it designed which allows for outdoor inspection. It is suited to detect all type of volumetric flaws such as corrosion, wear, cracks, and porosities. It may provide good sensitivity to small flaws at or near the surface of the sample. The part under inspection doesn’t require much preparation while testing using the eddy current technique. As any other non-destructive techniques, there are some disadvantages associated with eddy current technology.

The limitations with the eddy current testing technique are:

- The technique is very susceptible to any changes in the magnetic permeability (μ) and conductivity (σ) of the material.; any type of changes is shown as a false defect signal because of the disturbance of the eddy currents distribution, especially in ferromagnetic materials.
- Due to the way eddy currents is created, this technique is only effective on conductive material and the material must be able to support a flow of electrical current.
- Another constraint is that it won’t be able to detect defects that are parallel to the surface since the flow of the eddy currents are always parallel to the surface, if there is a planar defect that does not interface with the eddy current then the defect won’t be detected.
- In addition, if the surface roughness of the test part is large, it will interfere with the resultant signal [13,14]. The defect detection in parts made by AM processes using eddy current technique depends on the grain structure and surface finish of the part under test. There are some ways to filter any background noise or ripples in the result signal by applying filters to it or by machining the surface of the test part to smooth it out and arrive at a better detected signal.

**Figure 2:**
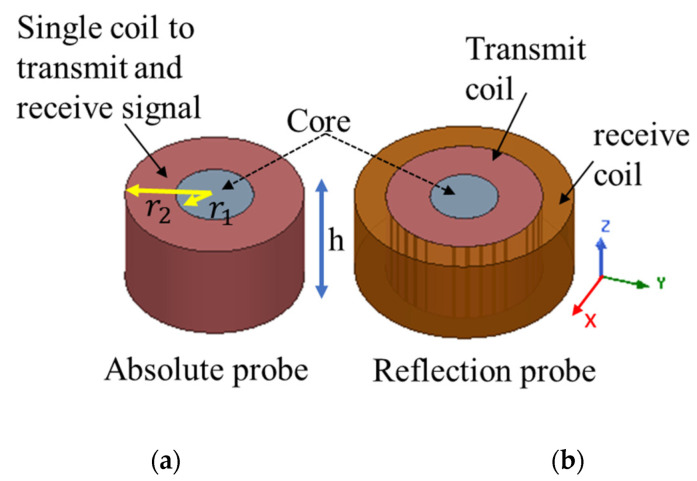
Absolute and reflection probe geometry differences; (**a**) shows an absolute probe that has only one coil, (**b**) shows a reflection probe that has two coils.

The main tool used in eddy current testing is the probe used for the detection. Eddy current probes are responsible for inducing the eddy current inside the material and in the meantime detecting the defect signal. There are different types of eddy current probe geometries. All types of probes can locate the defects but only certain probes with different coil orientation can be used to give an estimation about the defect size. Most of the available probes either has one coil which works in the absolute mode or two coils which work in the reflection or differential mode. Figure 2. shows the geometrical difference of absolute and reflection probes. The smaller the diameter of the probe the smaller size of the detectable defect.

In this paper a comparison will be carried out between the response of an absolute designed probe and a reflection commercial probe for subsurface defects in parts made by laser powder bed fusion technology. Comparison between different eddy current probes with different coil specs helped understanding the effect of each parameter such as coil length, inner diameter, outer diameter, number of turns, and the different operating modes on the probe sensitivity to detect cracks with width less than 0.3 mm and voids with diameter less than 0.3 mm. Exploring both probes performance towards some of the limitations and challenges that face using eddy current testing technique in additive manufacturing such as surface roughness is carried out by testing different samples with different surface roughness sizes in the range of 8 μm to 11 μm.

In this paper, main contribution is shed some light on the use of eddy current NDT technique for the defect detection in additively manufactured components. There are not many studies on the application of ED in AM and the authors intend to fill this gap. Another contribution is the proposing of a method to reduce the signal sensitivity to the surface roughness and the edge effect for better detection of flaws inside components that will be discussed in the sensitivity analysis section. An introduction about additive manufacturing and eddy current technique is provided in Section 1. Type of samples used for testing by both probes and the specs for the probes are explained in Section 2. Testing the designed absolute and the commercial reflection probes on different samples made of different materials with different flaws is described in Section 3. A comparison between each probe response to the defect is provided in Section 3. Sensitivity analysis provided in Section 4. Finally, conclusion and future work are listed in Section 5.

## 2. Materials and Methods

### 2.1. Overview

One of the probes used for comparison is an absolute type that has one coil to induce the eddy currents inside the material and detect the defect signal. The absolute probe dimensions are listed in Table 1. The other probe used for comparison is a commercial probe that works in the reflection mode. The reflection probe has two coils, one coil transmits eddy currents inside the material and the other coil receives the detected defect signal.

Frequency used for the commercial probe which works in reflection mode is 2 KHz. The peak-to-peak voltage value supplied to the probe is 12 V. Its diameter is around 9 mm which is almost half the diameter of the absolute probe. Using a current probe, the measured RMS input current value is 0.0205 AMP. The length of the probe is around 35 mm.

### 2.2. Samples Used for Experiment

Some of the common materials that are used to build products by laser additive manufacturing are stainless-steel, titanium. Stainless-steel and titanium have high conductivity and magnetic permeability. They are used in several applications such as customized products include artificial joint or bone replacements, instruments for surgery or prosthetics [15]. In addition, both materials have been widely used in Aerospace applications such as jet engine components [16,17].

Samples used for experiment to test both probes are made of stainless-steel and titanium. The stainless-steel (SS316L) samples were printed using a Renishaw AM400 machine and the titanium (TI64) samples were printed using EOS M290 machine. The process parameters used during printing the stainless-steel samples are hatch power 190 W, hatch distance 60, borders power 140 W, borders distance 30, fill hatch distance 0.110 mm, and repetition limit 20. The process parameters used during printing the titanium samples are stripes distance 0.09 mm, stripes speed 1250.0 mm/s, stripes power 195.0 W, beam offset 0.015 mm, stripe width 5 mm, and stripes overlap 0.12 mm. All samples have subsurface artificial defects and are designed using Solidworks software.

**Figure 3:**
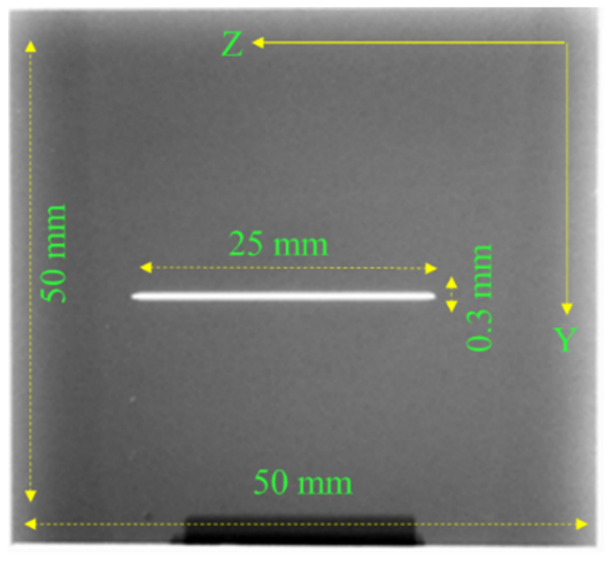
CT results of a stainless-steel sample.

To be able to check the accuracy of the defect width, depth and length, CT has been used as a way to check each defect dimensions. All stainless-steel and titanium samples inspected using CT. In Figure 3 it shows one of the stainless-steel samples used for experiment which has a subsurface notch with 25 mm length and 0.3 mm width. The notch is 1.5 mm below the surface and is not visible visually. Images were taken using a ZEISS Xradia Versa 520 at the following settings:

- Voltage: 140 kV.
- Power: 10 kW.
- Exposure Time: 2.0 s.
- Source Distance: 110.2702 mm.
- Detector Distance: 27.0012 mm.
- Pixel Size: 55.1493 μm.
- Optical Magnification: 0.39328.

**Figure 4:**
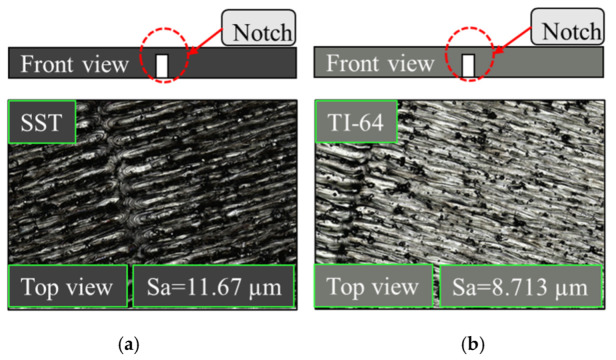
The surface roughness size for both materials where (**a**) stainless-steel (316) sample, (**b**) Titanium sample (TI-64).

All stainless-steel and titanium samples have rough surfaces because of the laser hatch pattern and the random distribution of the powder. Surface roughness is one of the challenges that makes it difficult to interpret the measured signal. The surface roughness and finish of the test piece will cause disturbance to the flow of the eddy currents inside the material which produces unwanted signal in addition to the actual defect signal. Detecting small size defects in parts with high surface roughness will be difficult and requires applying different filters to the measured signal. An example of the different surface roughness in stainless-steel and titanium parts made by additive manufacturing shown in Figure 4. The surface roughness size of all samples was measured using the microscope as shown in Figure 4. The mean of parameter (Sa) in Figure 4. is generally used to evaluate surface roughness, it expresses, as an absolute value, the difference in height of each point compared to the arithmetical mean of the surface. The images were taken using a Keyence VK-X250 Laser Con-focal Microscope with the following settings:

- 20× Magnification Lens.
- 0.5 μm Z-pitch.
- 693.630 μm X-Y calibration per pixel.

All samples’ sizes are 50 × 50 mm. The defects are located at the center of each sample to be able to move the probe easily and to avoid edge effect [18] or perturbing the eddy currents.

### 2.3. Simulation for Notches and Voids

**Figure 5:**
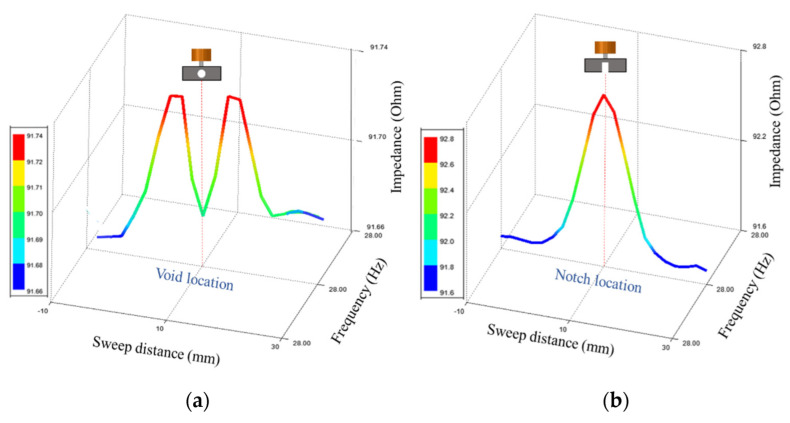
Shows simulation results of the probe impedance change on top of a plate with a defect at 10 mm, where (**a**) defect type is a sphere, (**b**) defect type is a notch.

There are two cases considered for simulation to show the difference of the probe response in case of a notch and in case of a void. The parameters for the simulation model shown in Table 2. The sweep direction of the coil is in the Y direction and the defect is located at 10 mm position as shown in Figure 5. The step size used for the parametric sweep in 2 mm. The probe gives a different response in case of a notch compared to the void. There are different mesh types that are supported by ANSYS Maxwell, eddy current solution such as inside selection (length based) and on selection (length based or skin depth). The mesh type used during simulation is on selection, skin depth based, number of layers of elements is 2. The surface triangle length is 0.2 mm.

In Figure 5, the types of defects detected are a sphere that simulates a subsurface void and a notch that simulates a subsurface crack. Simulation carried out using same input values and dimensions of the absolute coil. The sweep distance is 40 mm, and it starts from 10 mm before the zero position to 30 mm after the zero position. In Figure 5a,b, the magnetic field will be the minimum when the coil’s center is aligned with the defect’s center and there won’t be much change in the coil impedance. The magnetic field will reach its maximum when the coil winding is centered over the defect causing a big change in the coil impedance. In case of a sphere, the defect will still give maximum change in the field in as the sphere largest cross-section will intersect with the field at all orientations. In case of a notch, the changes in the coil impedance will only happen if the notch is perpendicular to the electric field. Some examples that show eddy current imaging for defect characterization can be found in [19]. In Figure 5a, the void diameter is 3 mm, which is big, and that will cause generating two different peaks when the edges of the coils are aligned with the edges of the void during a sweep motion.

### 2.4. Analytical Modeling

Eddy currents can go deeper in the material for a certain depth. The penetration depth is determined by the material conductivity, permeability and the frequency used for the source current. The standard depth of penetration formula is shown in (1).

(1) $$\delta \approx \sqrt{\frac{2}{\mu \sigma \omega }}$$

where, δ is the standard depth of penetration (SDP), σ is the material conductivity, ω is the excitation frequency, and μ is the material magnetic permeability of the test piece [20]. The eddy current governing physics can be expressed mathematically in a diffusion equation form. It can be described mathematically using (2) based on the vector potential **A**.

(2) $$\nabla^{2\;}A+j\sigma \omega \mu A=-{\mu \;J}_{\mathrm{source}}$$

where, J is the current density of the source excitation coil and $\nabla^{2\;}$ is the Laplace operator [21]. The Dodd and Deeds model gives a closed integral form solution of the vector potential [22,23]. There are some other analytical methods that are used to solve the eddy current problems such as using the TREE method.

There are different analytical models that can be used to calculate the coil impedance change on top of plate with a defect. Some of these models uses the integral form and others uses the truncated region eigenfunction expansion (TREE) method. The impedance value of the probe on top of a plate made of stainless-steel and titanium materials without a defect is calculated using (3) as mentioned in [24]. The coil impedance magnitude value calculated analytically on top of a stainless-steel plate is 81.8 ohm. The coil impedance magnitude value calculated analytically on top of a titanium plate is 83.9 ohm.

(3) $$\Delta Z=-j2\omega \pi {ϻ}_{0\;}b^{5}\overset{\infty }{\underset{m=1}{\sum }}L_{\mathrm{Nm}}C_{m}{}^{2}\exp\left(-2x_{m}l\right)$$

where $C_{m\;}$ can only be determined by inner diameter ($r_{1}$), outer diameter ($r_{2}$), number of turns (w), and coil height (h). The complex function $\left(L_{\mathrm{Nm}}\right)$ is determined by the plate thickness, its material conductivity, and relative permeability. The Lift-off distance (l) and the truncated field region (b) [25]. Some of the values are shown in Table 1.

As mentioned before, to calculate the probe impedance value on top of a conductive plate with a flow, some models use the integral form that is based on the Dodd and Deeds model which solves the vector potential using the integral form in (4) [26].

(4) $$z=\left[\frac{3}{2}{\sigma \omega }^{2\;}{\left(\frac{A}{I}\right)}^{2}\right]\times {\mathrm{Vol}\alpha }_{22}$$

The term ${\mathrm{Vol}\alpha }_{22}$ depends on the defect geometry. In [27], the concept of truncated region eigenfunction expansion (TREE) is used to calculate the change in the coil impedance by truncating the solution domain to be finite. The main steps to calculate the coil impedance is to calculate the vector potential at different regions around the coil, under the coil and inside the material.

## 3. Measurement Results and Discussion

### 3.1. Measurements, Sensing and Circuitry

**Figure 6:**
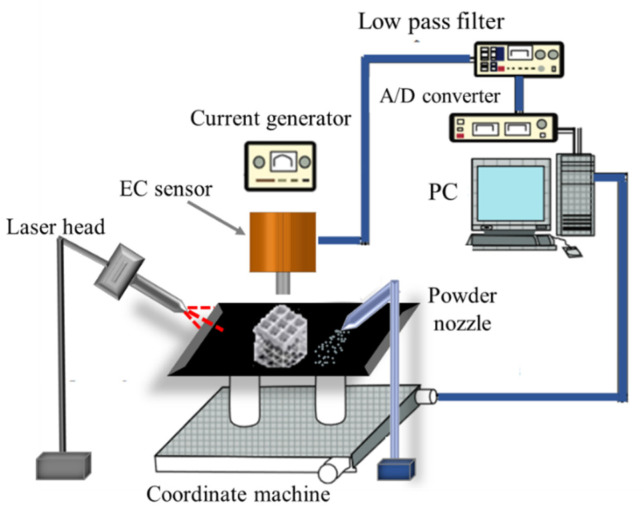
Schematic diagram of the defect detection and measurements during PBF process.

A schematic diagram of the defect detection while the parts are printed using LPBF technology shown in Figure 6. In the schematic diagram, the system has a current generator and a low pas filter, and A/D converter. Most of the data acquisition systems that exist in the market have these features. An alternating current or an alternating voltage is required to drive the probe coils to produce the magnetic field inside the coil. The supplied AC voltage to the absolute probe has a peak-to-peak value of 5 V and frequency of 28 KHz. The supplied peak-to-peak voltage value to the reflection probe is 12 V and the frequency used is 2 KHz. Based on the used frequency and both stainless-steel and titanium conductivity and permeability, the eddy currents standard depth of penetration is around 3 mm inside the material.

There are two different factors that help reduce the effect of the noise produced by the subsurface. First, applying a low pass filter to the detected signal. Second adding a thin coating layer to the bottom of the probe. Applying a filter is necessary to reduce any noise in the measured signal and to differentiate between the actual defect signal and the noise. Applying a filter helps to remove the background drift from the eddy current signal. Different types of filters can be applied to the measured signal such as low pas filter, high pass filter, band-pass filter, and median filter. Choosing which filter should be applied to the measured signal depends on the length of the flaw and the noise source. The length of the filter should be at least twice the length of the defect otherwise the defect signal will be attenuated. The filter type that was used during measuring the defects signals which are shown later in Section 3, was a low pass filter with a cutoff frequency of 30 Hz. The low pass filter passes only signals with low frequencies and cuts off high frequency signal such as electrical noise. The PC is used to control the whole system and the detection process. The data acquisition system used during the detection process is MIZ-12C. To move the probe on top of the samples in different direction, a 3D printer XYZ Motorized Linear Stage could be used which will help keeping the lift off distance between the probe and the sample constant.

**Figure 7:**
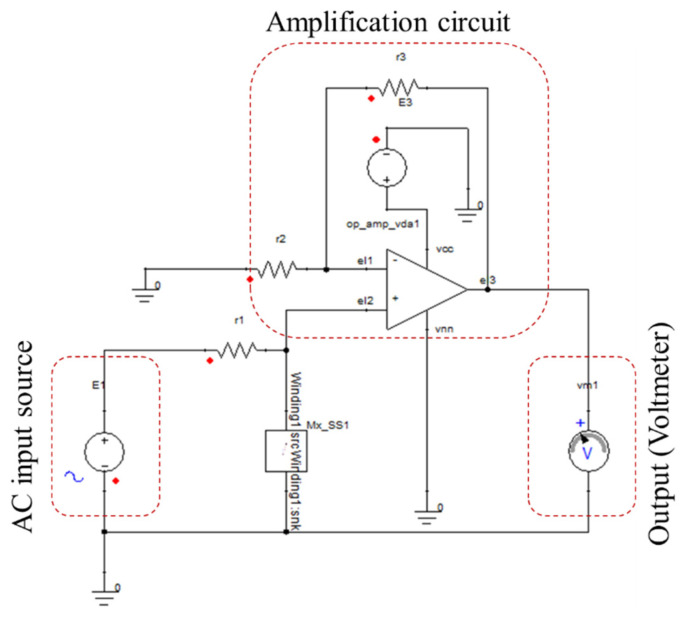
Circuit attached to the absolute probe.

An illustration of the circuit attached to the probe for the experimental setup is shown in Figure 7. To drive the absolute probe coil, a simple RL circuit was attached to it. The coil winding is attached to the voltmeter to be able to measure the flaw signal and attached to an AC voltage source to be able to drive the coil and induce eddy currents inside the part under test. The peak-to-peak voltage value supplied to the probe’s coil is 5 V with a frequency value of 28 Hz. The coil winding is attached to a resistor (R1) with a value 27 ohm. An operational amplifier used to amplify the measured signal since the measured signal value is in millivolts. The op-amp type used is LM358 and 5 volt is supplied to the operational amplifier. The non-inverting amplifier used has an input resistance (R2) with a value of 1000 ohm and an output resistance (R3) with a value of 10,000 ohm. The calculated gain of the op amp is 11. The circuit provided in Figure 7 is the one provided to the simulation model created in ANSYS Simplorer to be able to validate the experimental results.

**Figure 8:**
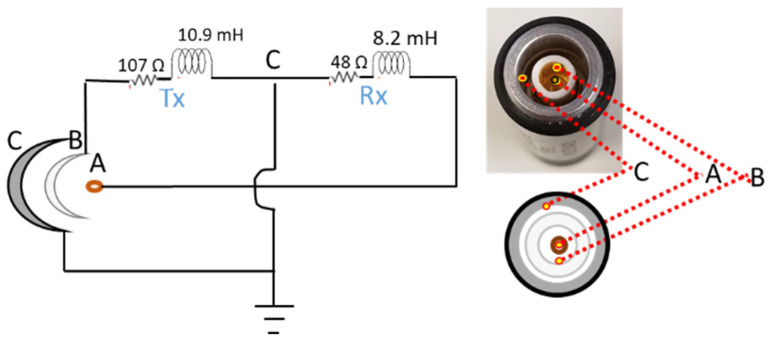
An illustration of the reflection probe circuit.

The circuit attached to the reflection probe is shown in Figure 8. It consists of two parts, the transmitter part that is attached to the transmitter coil and the receiver part that is attached to the receiver coil. The peak-to-peak voltage value supplied to the transmitter coil is 12 V with frequency of 2 KHz. The receiver coil is attached to a voltmeter to measure the defect signal. The measured inductance value of the transmitter coil is 10.9 mH and the measured inductance value of the receiver coil is 8.2 mH. Both absolute and reflection probes are connected to the data acquisition equipment using a Triax cable and a LEMO connector type where A, B, and C are the connection points. An illustration of the reflection probe circuit and the LEMO connector attached to it is shown in Figure 8.

**Figure 9:**
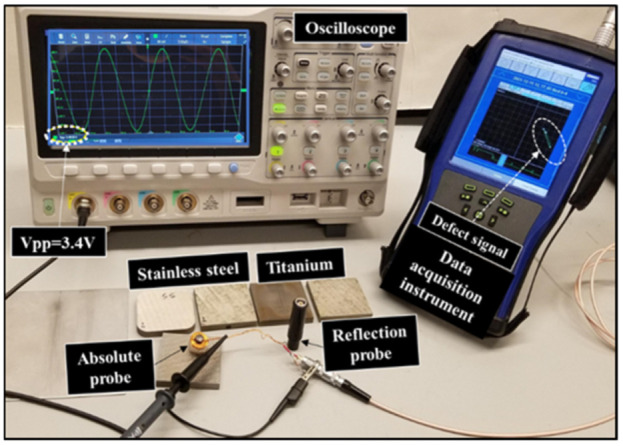
Experimental setup showing both probes used to test titanium and stainless-steel samples.

To be able to compare between both probes, samples made of stainless-steel SST-316 and titanium TI-64 were used for the detection process. Multiple readings taken for each defect using both probes. Since both probes have different dimensions and different input values, the measured signals were normalized for comparison. Each defect has six readings, and the normalized value was calculated by dividing each voltage of the six reading by the maximum voltage value (Vpp) of all the six readings [23]. The defects are located at the center of each sample. The samples dimensions, defect size and location are shown in Figure 3. The experimental setup used in this work is shown in Figure 9. The measured peak-to-peak voltage at the probe terminal on top of a stainless-steel sample is 3.4 volt using the oscilloscope. The RMS input current value is 0.0148 Amp. The input Vpp to the coil is 5 V and the resistance connected in series with it is 27 ohms. The measured value of the probe impedance on top of a stainless-steel plate and titanium plate using the absolute probe can be calculated using (5).

(5) $$Z=\sqrt{R^{2}+x_{l}{}^{2}}=\frac{V}{I}$$

where V is the measured Vpp value of the probe on top of the stainless-steel or the titanium plates. The RMS value of the measured Vpp is 1.202 V. Using Equation (5), the measured coil impedance is 1.202/0.0148 = 81.2 Ω.

### 3.2. Detecting Defects on Stainless-Steel Samples

Different defect types can be formed in parts made by AM technology such as cracks and porosities due to lack of fusion. Lack of fusion porosities are formed of unmelted powder particles and usually have irregular shapes [28]. The size of porosities is usually around 5–200 µm. Cracks with the range between 200–700 µm are formed in different alloys (e.g., superalloys) due to the great residual thermal stress combined with the high temperature gradient [24,25,26,27,28,29].

Several cases were considered for testing both probes. The first case is to detect 0.07 mm, 0.1 mm, 0.2 mm, 0.3 mm and 0.4 mm notches width. All notches have a length of 25 mm and located at 1.5 mm from the sample surface. All samples thickness is 8 mm. Samples are made of stainless-steel (316) with conductivity 1.33 MS and relative permeability 1.01. To simulate subsurface cracks, the detection process is carried out form the opposite side of the notches.

Second case considered for testing both probes is to detect blind holes which are located at 0.5 mm from the surface with different diameters. The diameters are 0.34 mm, 0.4 mm, 0.54 mm, and 0.6 mm. The samples thickness is 2 mm.

**Figure 10:**
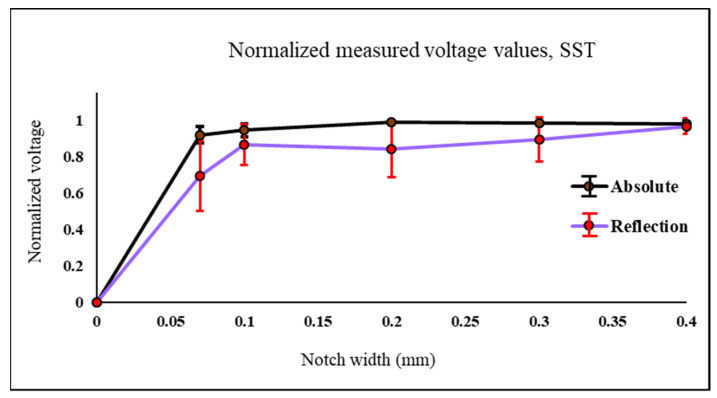
Normalized values of the measured voltage for both probes on top of plates made of stainless-steel with notches with different widths.

**Figure 11:**
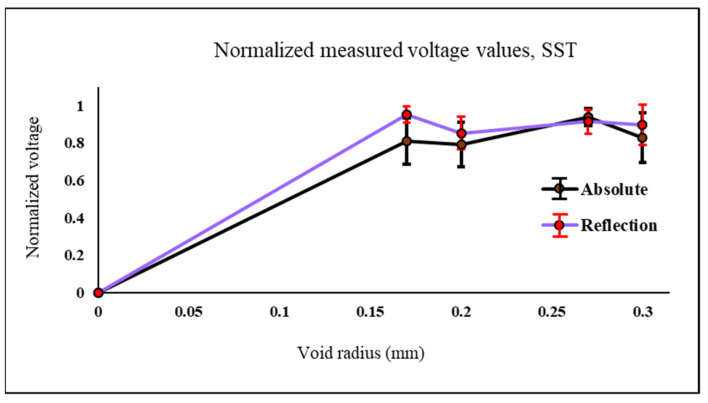
Normalized values of the measured voltage for both probes on top of plates made of stainless-steel with different radius blind holes.

**Figure 12:**
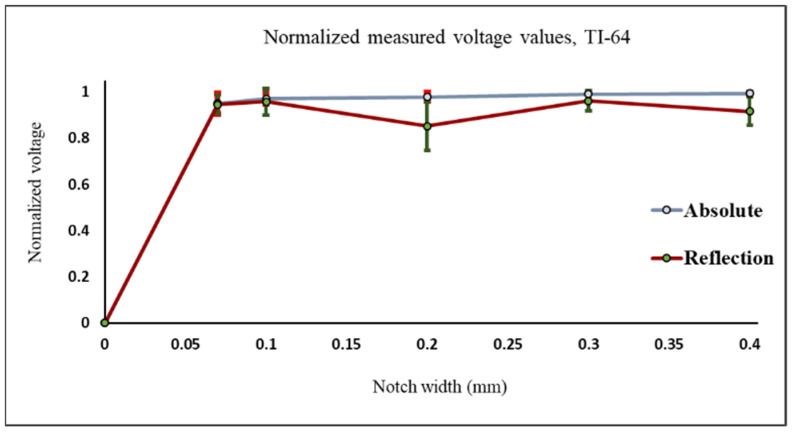
Normalized values of the measured voltage for both probes on top of plates made of titanium with notches with different widths.

**Figure 13:**
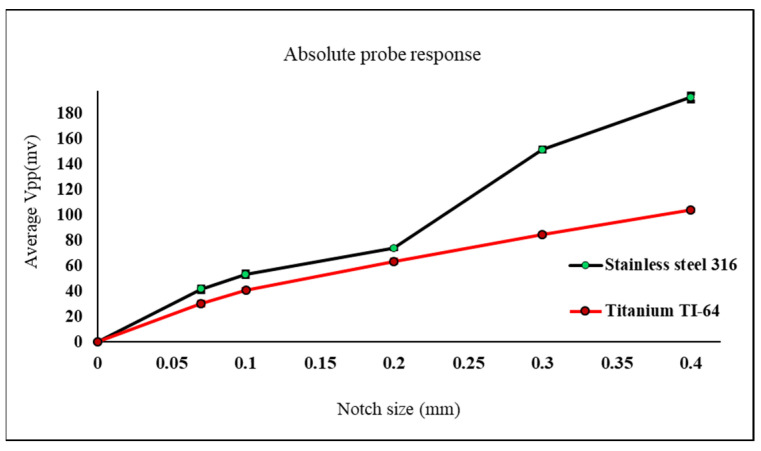
Measured Vpp values using absolute eddy current sensor on top of titanium and stainless-steel samples with notches.

**Figure 14:**
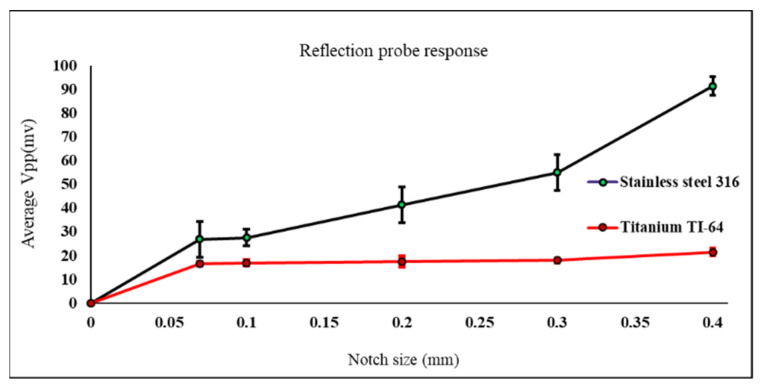
Measured Vpp values using reflection eddy current sensor on top of titanium and stainless-steel samples with notches.

Values shown in Figure 10 and Figure 11 are the ones obtained experimentally. An example of the samples used for experiment shown in Figure 3. The probe is moved across each defect in the Y direction. In Figure 10 both probes are used to test the stainless-steel plate with notches and the measured signal is the raw peak to peak voltage measured at the probe terminals in millivolts. Values shown in Figure 10, Figure 11 and Figure 12 are the normalized value. The actual peak to peak voltage of the defect signal using both probes are shown in Figure 13 and Figure 14. As shown in Figure 10 the absolute probe shows a better response detecting the notches compared to the reflection probe. There is a relationship between the size of the detected defect and the diameter of the probe coil. The smaller the diameter of the coil the smaller the defect that can be detected. Since the reflection probe coil has a diameter that is almost half the diameter of the absolute probe coil, it gives a better response detecting small size voids, meanwhile the absolute probe gives a higher response and higher measured signal value in case of notches since the obstruction of the eddy current flow is higher because of the bigger diameter size.

In Figure 11. the reflection probe gives a better response detecting the blind holes compared to the absolute probe since the diameter of the reflection probe is almost have of the absolute one which improved its detectability to smaller defect sizes. The average of all normalized values for each defect and the standard deviation error bars are the one shown in Figure 10 and Figure 11.

### 3.3. Detecting Defects on Titanium Samples

Third case considered for testing both probes is to detect notches with same width and length such as the ones used in the first case. The samples are made of titanium and the notches are located at the center of the sample. An example of the samples shape is shown in Figure 3.

The samples are made of titanium (TI-64) with conductivity 0.56 MS and relative permeability 1.00005. Both probes were used to test the samples made of titanium. The measured peak to peak voltage of the defect signal was normalized and plotted as shown in Figure 12. The titanium plates have notches with the same length of 25 mm and each notch is located at the center of the plate as shown in Figure 3. The absolute probe gives a higher and better response to each notch compared to the reflection probe. The repeatability of the measured voltage values of each notch using the absolute probe is also better and the average values are the ones used in Figure 12.

### 3.4. Material Conductivity Comparison for Both Probes

Another comparison conducted using both probes, shown in Figure 13 and Figure 14, exhibits how much the detected defect signal will be affected in case of testing different materials with different conductivity. The comparison carried out based on the measured defect signals obtained from the samples used in the first and third cases.

Materials with higher conductivity will cause higher amount of eddy currents to be induced inside the material. Therefore, the existence of any crack inside the material with the higher conductivity will perturb these eddy currents causing a higher probe response and a higher measured voltage value.

In Figure 13, a comparison on the absolute probe detection response to notches on stain-less steel and titanium samples is carried out. The measured Vpp values at the probe terminal in case of stainless-steel is higher than the measured ones in case of titanium since stain-less steel (316) has a higher conductivity than titanium (TI-64).

Same behavior found using the reflection probe during testing both materials in Figure 14. The probe is moved across each defect multiple times keeping the lift off distance constant all the time. Looking at Figure 13 and Figure 14 the error bars shown for each defect represent the repeatability of the measured peak-to-peak voltage values for all six readings for each defect. In Figure 13 the six readings of the measured peak-to-peak voltage for each defect show more repeatability which means that the results are more accurate and reliable compared to the results shown in Figure 14. Based on the previous explanation, the absolute probe shows better results to detect notches compared to the reflection probe.

## 4. Sensitivity Analysis

In this section different scenarios are considered for sensitivity analysis. The first case is to detect defects that are close to an edge. The second case is to detect multiple defects that are located in the same region or at a close distance from each other.

### 4.1. Testing Close to an Edge

**Figure 15:**
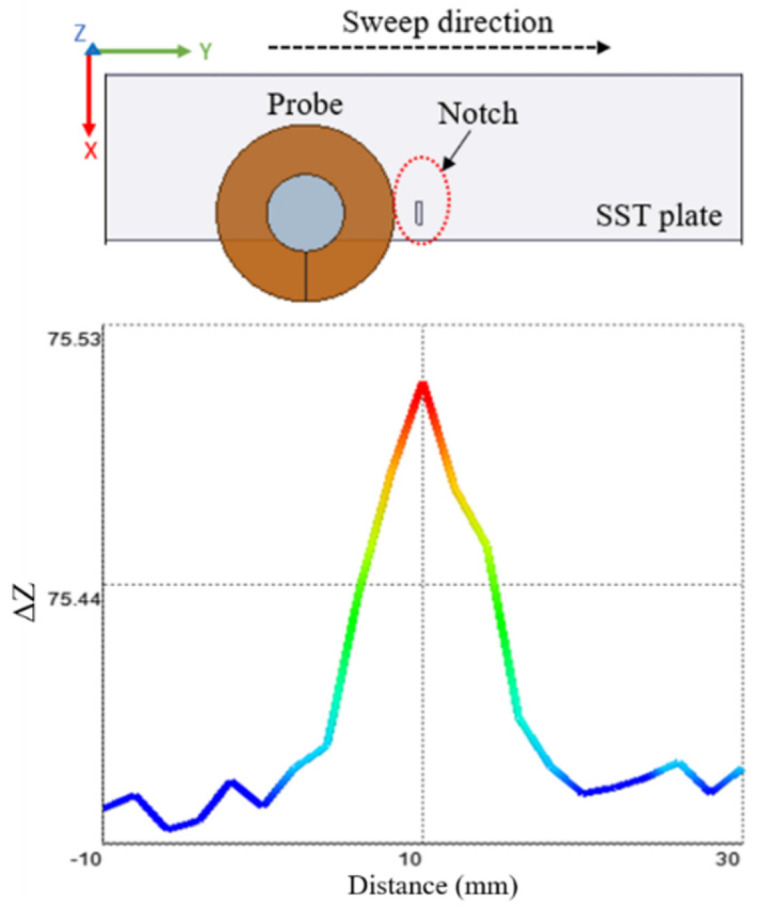
Subsurface notch close to an edge.

The possibility of detecting subsurface defects that are close to an edge are explored by creating a simulation model that have subsurface notch. Moving the probe parallel to the edge of the plate helps detecting the defect without acquiring a false signal of a flaw that represents the edge of the sample, as shown in Figure 15. The subsurface notch length is 2 mm and 0.3 mm width. Plate thickness is 4 mm, and the defects are located at 1.5 mm under the surface.

### 4.2. Multiple Defects in the Same Region

**Figure 16:**
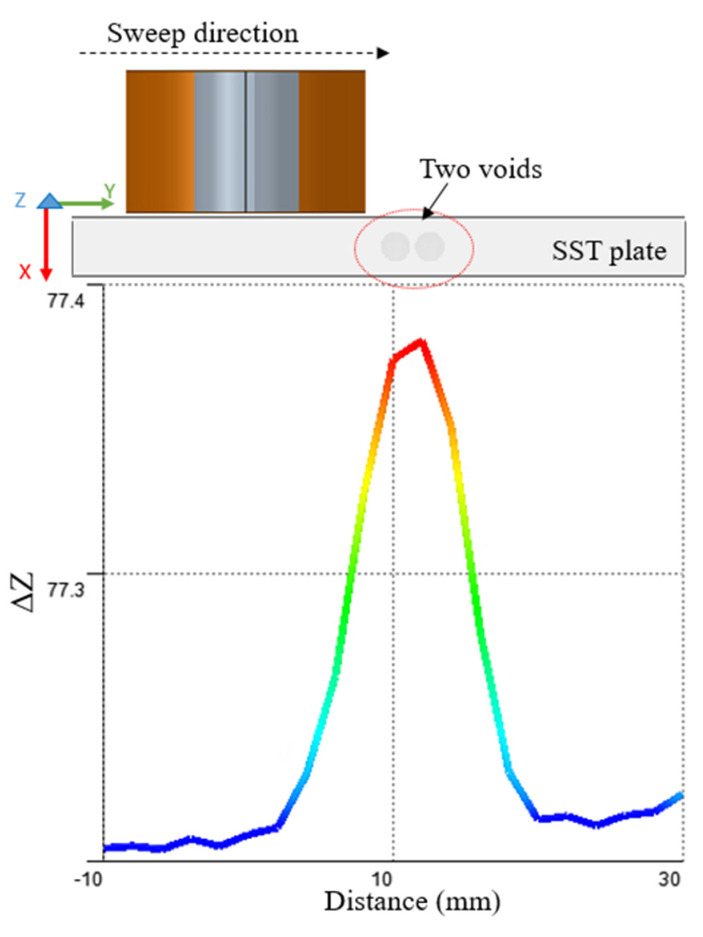
Sweep over position on the top of the plate with two voids besides each other in the same region.

**Figure 17:**
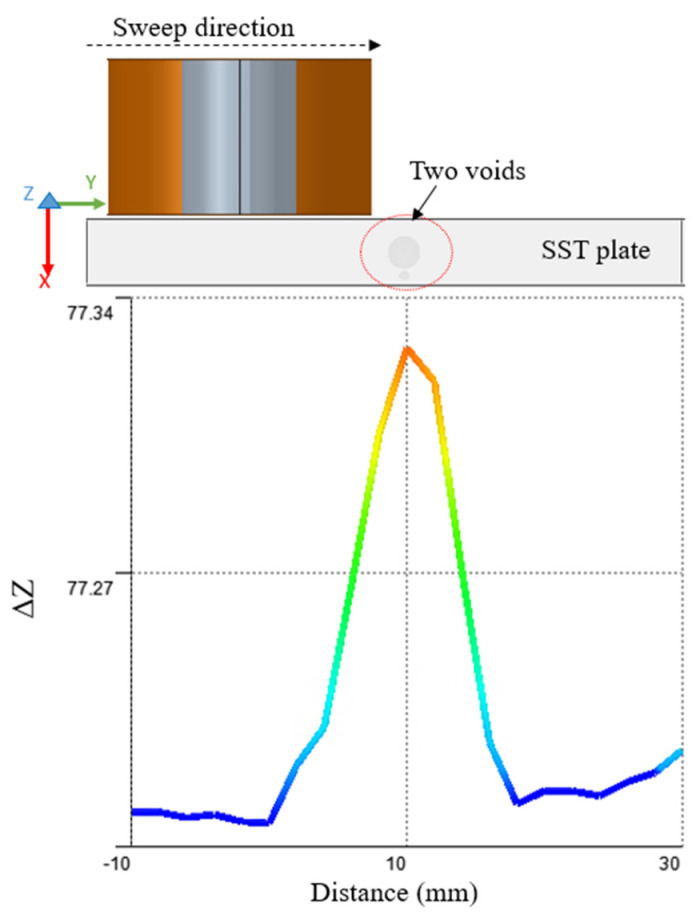
Sweep over position on the top of the plate with two voids above each other in the same region.

The second case is to explore detecting multiple defects that exist in the same region or defects that are separated by small distance. There are two different cases considered in this analysis. The first case is to have two voids parallel to each other (Figure 16) and the second case is to have two voids on top of each other (Figure 17). In the first case, where there are two voids that are almost the same size, located close to each other in the same region, the peak value of the impedance change is affected by both defects. It gives one peak value change, while moving the probe on the top of the plate, instead of showing two different peaks for each void.

In the case, where there are two voids with different sizes that are located on top of each other in the same region, the peak value of the impedance change is affected by both defects. This configuration gives one peak value change in case of moving the probe on the top of the plate instead of showing two different peaks for each void. The peak normally represents equivalence of both the bigger size void and the smaller one. In Figure 16, there is only one peak shows in case of two voids parallel to each other because the space between them is too small. In case the space between both voids (in the Y direction) is big enough which depends on the coil geometry and the eddy current flow under it, it will show two different peaks for each void. In Figure 17, there is only one peak shows in case of two voids on top of each other because both voids center are aligned regardless of spaces between them.

## 5. Conclusions

A comparison carried out between the responses of two different probes with different geometries which work in different modes to detect defects in samples made of titanium and stainless-steel. The samples are made by AM using laser powder bed fusion with artificial defects. In addition, different defect shapes were detected such as notches that simulates subsurface cracks and blind holes that simulates subsurface porosities. Another comparison carried out using both probes to compare the effect on the defect signal from the point view of conductivity. The absolute probe gives a better response in case of detecting notches meanwhile the reflection probe gives a better response in case of detecting blind holes. The absolute probe is more suitable to detect cracks and incomplete fusion holes. The reflection probe is more suitable to detect small diameter blind holes with diameter less than 0.2 mm. It is easier to design and fabricate the absolute probe and cheaper to implement it. The minimum defect size that can be detected using the designed absolute probe applied to the experimental configuration used in this paper is 0.2 mm void radius and 0.07 notch width. For future work, random defects with unknown sizes in the range of 0.05 mm to 0.5 mm width and different lengths in the range of 1 mm to 5 mm can be tested to acquire its approximate size. In addition, detection of defects same size at different depths in the range of 1 mm to 3.5 mm can be explored.

## Figures and Tables

**Table 1:** Coil and Core Parameters of the Absolute Probe.

Coil outer radius $(r_{2}$)	8 mm
Coil inner radius $(r_{1}$)	3.5 mm
Coil length (h)	9.435 mm
Core diameter	7 mm
Core length	13.435 mm
Input current	0.0148 RMS AMP
Wire gauge	AWG-25
Diameter of the wire	0.45466 mm
Frequency (f)	28 KHz
Voltage	5 V
Plain core type	Ferrite
Coil inductance	444.942 μH
Number of turns (w)	164

**Table 2:** Parameters Used for Both Simulation Cases.

Plate thickness	8 mm
Plate length	50 mm
Material	Stainless-steel (316)
Notch length	15 mm
Notch width	0.4 mm
Void radius	1.5 mm
Lift off distance	0.2 mm
Defect location	10 mm in Y direction

## Data Availability

Data available from the authors upon request.
